# Local Anesthesia

**Published:** 1905-08

**Authors:** 


### Local Anesthesia.

Capt. J. W. Houghton, H. M. Army Medical Corps (*Journal of the Royal Army Medical Corps,* April 15, 1905,) says that Barker's method of effecting local analgesia by means of eucain-adrenalin solution gives most satisfactory results. He has lately used it for removal of a cystic tumor on the periosteum of the scalp; for removal of a fibroma on the posterior aspect of left trochanter, involving dissection from the bone; for opening a knee joint and removing loose cartilage; for excision of five varicose veins; for excision and ligature of external hemorrhoids in a case where chloroform was inadmissible; for opening and draining abscess of liver; for varicocele; and for laparotomy for perforating enteric ulcer. In all these operations the patients were free from pain, save the one with scalp tumor which was probably insufficiently infiltrated. Some patients knew when the knife was cutting them, without, however, feeling it as pain.

Barker's formula is:

| | | |
|---|---|---|
| Beta-eucain | 3 | grains. |
| Sodium chloride | 12 | " |
| Distilled water | 3½ | ounces. |
| Adrenalin chloride (1.1000) | 18 | drops. |

The whole quantity may be injected at one time, but one-third of it usually suffices. The adrenalin should be added only when the eucain solution has been boiled and cooled. Infiltration of this solution causes a complete analgesia lasting several hours, the anesthetic effect of the eucain being enhanced and lengthened by the adrenalin, which actively contracts the arterioles, limits the blood supply, and retains the eucain in position.

Houghton sees no reason why amputations and hernia operations cannot be performed by the method, the main factor being the infiltration of all nerve trunks supplying the site, not forgetting the probability of nerve anastomosis. The more one sees of this analgesia, he remarks, the more is it preferred to general anesthesia, the patient escaping the risks and after-effects of the latter and the operator being saved anxiety.

